# Ignaz Semmelweis: a victim of harassment?

**DOI:** 10.1007/s10354-020-00738-1

**Published:** 2020-03-04

**Authors:** Sonja Schreiner

**Affiliations:** Institut für Klassische Philologie, Mittel- und Neulatein, Philologisch-Kulturwissenschaftliche Fakultät, Universität Wien, Universitätsring 1, 1010 Vienna, Austria

**Keywords:** Botanical doctoral thesis, Puerperal fever, Multilingual correspondence, Comparative physiology, Afterlife in literature, film, and drama

## Abstract

Ignaz Semmelweis’ (1818–1865) discovery of the endemic causes of *febris puerperalis* is a striking example of the role of pathology in medicine. Transdisciplinarity encounters Semmelweis’ biography, which is neither linear nor totally focused on medicine. He completed the *philosophicum* (*artisterium*), studying the *septem artes liberales* (1835–1837) in Pest, comprising humanities and natural science. After moving to Vienna, he began to study law, but turned to medicine as early as 1838. In 1844, he graduated with a botanical doctoral thesis composed in Neo-Latin, showing linguistic and stylistic talent and a broad knowledge of gynecology and obstetrics. The style and topoi demonstrate the interchangeability of what he learnt during his *propaedeuticum*. Nowadays, hardly anyone is familiar with this booklet, for two main reasons: the language choice and the life-saving impact of the physician’s *opus magnum* on the reasons for puerperal fever (*Die Aetiologie, der Begriff und die Prophylaxis des Kindbettfiebers*). In later life, he became convinced that he had no talent as a (scientific) author—a fatal error that led him to become a victim of what we now call “publish or perish.” Semmelweis had felt rejected for years. This negative feeling was the reason for his decision not to publish his great book for 14 years. When it finally went to the printer in 1861, the scientific community did not accept it. This experience caused psychosomatic symptoms owing to his long-standing and deeply felt disappointment. Bad conscience tortured him. This permanent stress destroyed his health: in 1865, his relatives (including his wife) and friends took him from Budapest to Vienna. He thought he was going to spend some time relaxing, but in fact was led into a newly built asylum for the mentally ill, the *Niederösterreichische Landesirrenanstalt*. When he realized what was happening, he tried to escape. Badly abused, he died from sepsis caused by open wounds and a dirty straightjacket 2 weeks later. This article will show Semmelweis to be a multilingual author of scientific literature and (open) letters; it will present him as a researcher who became a victim of harassment and what is referred to as the “Semmelweis reflex” (“Semmelweis effect”); and it will focus on his afterlife in (children’s) literature, drama, and film.

## Optimistic (botanical) beginnings or: the Neo-Latin thesis of Ignaz Semmelweis

Ignaz Semmelweis’ (1818–1865) discovery of the endemic causes of *febris puerperalis* is a striking example of the role of pathology in medicine. Transdisciplinarity encounters Semmelweis’ biography, which is neither linear nor totally focused on medicine. He completed the *philosophicum* (*artisterium*), studying the *septem artes liberales* (1835–1837) in Pest, comprising humanities and natural science. After moving to Vienna, he began to study law, complying with the wishes of his father, a merchant, but turned to medicine as early as 1838. In 1844, he graduated with a botanical doctoral thesis^1^ composed in Neo-Latin, showing linguistic and stylistic talent and a broad knowledge of gynecology and obstetrics. Nowadays, hardly anyone is familiar with this booklet, for two main reasons: the language choice and the life-saving impact of the physician’s *opus magnum* on the reasons for puerperal fever, meticulously described in more than 500 pages.^2^

- **1818: **Ignaz Philipp Semmelweis is born in Buda
- **1835–7: **… Completes the *philosophicum* at the University of Pest
- **1837: **… Studies Law at the University of Vienna
- **1838: **… Skips Law and begins to study Medicine at the University of Vienna
- **1839–40: **… Continues his studies at the University of Pest
- **1841: **… Returns to Vienna
- **1844: **… Graduates with a botanical *dissertatio inauguralis*
- **1845: **… Becomes *doctor chirurgiae* and starts to work at the General Hospital
- **1846: **… Becomes assistant for obstetrics at the General Hospital
- **1847: **… Detects the causes of puerperal fever after the death of Jakob Kolletschka from sepsis
- **1849–50: **… Leaves for Pest, after the authorities failed to prolong his contract, where he successfully works in various hospitals for the rest of his life as a professor of gynecology and obstetrics, saving countless lives
- **1857: **Marriage to Maria Weidenhoffer, by whom he had five children (two boys and three girls, two dying as babies)
- **1861: **Publication of the *Aetiologie* and four open letters in two separate editions (each called *Zwei offene Briefe*)
- **1862: **Publication of the *Offener Brief*
- **1865: **Suffering from burnout, journey to Vienna, mysterious death

The year 2018, one of historical jubilees, was also one dedicated to Semmelweis: commemorating his 200th birthday, articles were published^3^ and symposia were held—without any focus on or analysis of his doctoral thesis.^4^ Knowing what happened later, and how he personally changed in response to the harassment^5^ he had to face, the first lines, i.e., a single sentence in his thesis (p. 3), are moving ones. He enthusiastically praises botanical plurality using metaphors taken from Pliny the Elder’s (†79AD) vast encyclopedia, the *Naturalis historia*:

*Qui oculis tam grate arridet amoenus foliorum viror, florum splendor et mira varietas; qui nares feriunt suavissimi odores, qui gustum demulcent dulcissimi succi, quae corpus nostrum restaurant, morbos profligant, sanitatem reducunt—substantiae plantarum, e quibus animum poetarum inspirat suavissimus Apollo *[…] *quam vim naturae—vitam—dicimus.* (“How brightly does the lovely green color of the leaves shine into the human eye. How delightful is the splendor of the flowers and their broad variety. How mild is their fragrance meeting the nose. How sweet is their taste—the substance of the plants restoring our bodies, fighting various illnesses, bringing back the healthy state, the ingredients out of which honeysweet Apollo inspires the poets’ souls, the thing we call the force of nature, the thing we call life.”)^6^

At the end of his encyclopedia, comprising 37 books, Pliny composes a prayer to the personified Mother Nature (§205). In analogy, Semmelweis presents the wide range of powers humankind can derive from flora.^7^ The style and topoi demonstrate the interchangeability of what he learnt during his *propaedeuticum*. In later life, he became convinced that he had no talent as a (scientific) author—a fatal error that led him to become a victim of what we now call “publish or perish.” Working at the traditional and catholic University of Vienna, while writing his thesis, he combined science and religion, a method professionalized decades earlier by the Jesuits, insisting on the driving force, the curiosity to find out the truth (p. 3): *Mens humana tamen non acquiescit, donec phaenomenorum omnium rationem reddat sufficientem, laeti ideo sequimur ideam, quam naturae philosophi hoc modo exponunt. Omne, quod existit, ex divino omnipotentiae spiritu emanat* […]. (“Nevertheless, the human mind does not rest until it finds a sufficient reason for all natural phenomena; that is why we happily follow the idea, the natural philosophers explain. Everything existing is a fruit of almighty god”).

## Semmelweis’ method of comparative physiology and his use of scientific language

Furthermore, Semmelweis worked systematically and gradually developed his theories, probably copying the familiar modes of exposition in the classes he had taken at university. He would do the same 17 years later in his book on fighting childbed fever, managing without perfect style, but convincing with figures, tables, and statistics. Traditionally, scientific literature in Latin was successful owing to its easily comprehensible terminology.^8^ Moreover, in Neo-Latin literature there are frequent reflections of the vernacular languages, e.g., p. 4: *Materia ex conflictu virium adtractivarum et repulsivarum resultat* […]*. Sunt vero potentiae hae: Calor, Lux, Aër, Electricitas et Solum. *(“Matter is a result of the conflict of attracting and repulsive forces. Those are the powers: heath, light, air, electricity, and ground.”) Semmelweis presents their necessity and distinguishes between *solum* (the ground as such) und *humus *(fertilizer). He shows the plants’ orientation towards the light, explained by the *heliotropium *(sunflower), by trees in the middle of the woods and at their margins, and by potatoes in the cellar seeking the sun coming in by a tiny window and thereby referring to an experiment in the footsteps of Goethe and Blumenbach.^9^

What follows is a lengthy chapter on reproduction, full of parallels to animals. Details close to embryology come in (p. 7): *Nucleus embryone et albumine completur. Embryo rudimentum novae plantae repraesentat; est ideo maxime essentialis seminis pars*. (“The nucleus consists of the embryo and the albumen. The embryo is the rudiment of the new plant, and at the same time the most essential part of the seed.”) Bulbs are *viviparae*, in other words: they give birth to living plants. When he writes a passage on *Organologia*, Semmelweis positions himself as a physician, using anatomical terms: the plants’ *vasa spiralia*, their vascular tissues, are equivalents to nerves (p. 12). Heaps of cells form organs, nutritional reservoirs are compared with fat cells in animals, and there are even male and female parts (pp. 24–5): *Pollen pulvis foecundans est*. […] *Ovarium ovula continet, quorum formationi ac evolutioni destinatum est; hinc animalium ovarium et uterum repraesentat*. (“The fertilizing powder is called pollen. […] The ovary contains eggs, destined to develop and evolve; it is comparable to the ovary and uterus of animals.”) The fragrance or odor of flowers are primarily interesting for health reasons (p. 23): *Spectato odorum principio chimico facile innotescit, cur flores in cubiculis adservati asthma, vertiginem, apoplexiam provocare valeant.* (“If you consider the chemical principle behind smells you will easily realize why flowers kept in bedrooms have the power to cause asthma, vertigo, and apoplexy.”)

Discussing monoecious and dioecious plants, he talks about hermaphroditism (p. 26): *Ceterum hermaphroditismum ceu typum in regno plantarum statuisse naturam arbitramur; nam sub certis tempestatis influxibus, scilicet extremis lucis et humiditatis, unum genus ab alio separari, masculinum in foemininum converti et contrarie immutari observamus. *(“Furthermore, to my belief, nature has given a place to hermaphroditism or gender in the reign of plants, since we observe that under certain influences of temperature, especially when light or humidity are extreme, one gender separates from the other, a male becomes a female one and the other way around.”) Strikingly enough, Semmelweis does not focus on animals that can do something similar (e.g., clownfish).

Finally, referring to the *Processus foecundationis*, he compares plants to amphibia and fish (p. 27): *ovula eum in modum foecundare, ut hoc in ranis, salamandris et piscibus noscimus*. (“Eggs are fertilized in the same way as we know from frogs, salamanders, and fish.”) Generally speaking, the elements and many species, including *homo sapiens*, help to reproduce plants (p. 30): *Semina leviora venti subinde per notabile spatium ferunt, graviora aqua per longum iter vehit, alia volatilia coeli dissipant, mammalia diducunt v. g. mures; quae tandem, homo hac in re faciat, quis non noscit! *(“Wind transports light seeds over a wide space, water delivers heavier ones over a considerable distance, the breeze of air brings flying ones from A to B, as do mammals, especially mice. And, last but not least, who does not recognize the important role of man in this respect?!”)

## Semmelweis’ exam and broad medical horizon

The last page of the booklet offers 10 *Theses defendendae *(“Theses to defend”), making a reconstruction of Semmelweis’ *rigorosum *possible. The starting point is the thesis itself: *Botanicae Studium pro Medico practico summi momenti *(“Botanical studies are very important for the practical physician”). What follows is based on Thomas Sydenham (*Non dantur morbi intermittentes—*“There are no intermittent diseases,” and *Causam hydropis melius principiis mechanicis quam dynamicis explicabis—*“You will better explain the causes of dropsy by mechanical principles than by dynamic ones”). The next thematic fields are pharmaceutics (*Sine Opio et Mercurio nollem esse Medicus—*“I would not like to be a physician without opium and mercury”), psychology (*Omnis Medicus sit Psychologus*—“Each physician should have psychological skills”), and anatomy, a Viennese “specialty”:* Fons floritionis medicinae modernae in Anatomia pathologica quaerendus est *(“The source of the flourishing modern medicine is to be searched for in pathological anatomy”). At the same time, here lay the roots of puerperal sepsis. Finally, Semmelweis had to discuss conservation (*Viget in omnibus corporibus nisus conservationis*—“In all bodies preservation is of high importance”), the importance of diagnostics (*Prognosis non de aegri, verum de Medici sorte decernit*—“Prognosis is decisive for the physician’s fate, not for the patient’s”), and pathognomony (*Nullum datur signum morbi pathognomonicum*—“There is no pathognomonic sign of illness”). Dangerous substances and venoms came at the very end: *Nullum venenum in manu medici *(“No venom in the doctor’s hand”). This sentence would become intriguingly important and subtle, especially to him—the pioneer of hand hygiene.

## The fatal effects of Semmelweis’ surroundings and intransigent character

Ignaz Semmelweis’ fate is inseparably tied to the “Second Vienna Medical School”:^10^ Carl Freiherr von Rokitansky, Ferdinand Ritter von Hebra, Josef von Škoda, and Jakob Kolletschka. When the latter died in 1847, Semmelweis was not in Vienna; but when he learned of the symptoms, he noticed the striking parallels to the women and their babies dying in childbed or in the first days of their young lives.^11^ An incautious student had cut his professor during an autopsy, which caused deadly sepsis.

The General Hospital housed two clinics specializing in obstetrics. Where the training of midwives took place, a significantly smaller number of patients died. Where students and professors worked, a huge number succumbed to childbed fever: the men went from the morgue to the women giving birth—without disinfection, since they did not know about bacteria. However, living people too could be vectors, not only cadavers—a fact Semmelweis realized only somewhat later. Deeply disappointed by the harassment he had suffered, Semmelweis in the meantime sulked and refused to publish his findings. His friends and supporters nonetheless tried to help disseminate them by word of mouth on his behalf. But when they did so, they unfortunately failed to mention the fact that living subjects, not just cadavers, could be infectious. This permitted his critics and opponents not to pay attention and to claim that there were no new and substantive findings.

When he finally, as extensively as savagely, wrote about his knowledge in his open letters of the early 1860s, it was too late.^12^ Nevertheless, he promptly (as early on as 1847) prescribed the washing of hands with chlorinated lime solution, a method that saved countless lives, but was unpleasant for the skin and seen as a waste of time by many. The established physicians of high reputation and not accustomed to new ideas could not accept that a young assistant (and a foreigner to boot) declared their hands to be unsanitary. Instead of respecting the convincing results and scientific progress, they exhibited a symptom called “Semmelweis reflex” (“Semmelweis effect”),^13^ a reaction that denotes denying new results out of principle. They preferred the old-fashioned *genius epidemicus*, atmospheric influence, climate theories, and the *miasma*, erroneous views going back to antiquity, followed and propagated by famous physicians of Semmelweis’ day. He criticizes this disturbing fact in his last publication of 1862, citing the Danish physician Thomas Bartholin who (in 1672) believed that childbed fever was more dangerous in autumn and winter owing to the climate influencing the uterus.^14^

It was Semmelweis’ fault that he attacked his “colleagues” in an inappropriate way:^15^ He called them murderers—even “efficient” ones—and Friedrich Wilhelm Scanzoni von Lichtenfels a “medical Nero.”^16^ It is no wonder that this wording caused enmity, even if it is understandable from the psychological point of view. Semmelweis had felt rejected for years. This negative feeling was the reason for his decision not to publish his great book for 14 years. In the meantime, he felt, and was in effect, relegated from Vienna to Budapest, where he continued his evidence-based methods with success. From an empirical point of view, he was definitely right. One has to add that he had left Vienna, since he refused to be reduced to demonstrations using the so-called phantom rather than real women.^17^ Semmelweis declared that he had been successfully fighting (for 14 years and in three hospitals) against *Pseudo-Puerperalfieber-Epidemien*,^18^ that statistics were on his side, and that his survival rates spoke a very clear language. Furthermore, he showed great familiarity with the scientific literature of the time by precisely citing and finding arguments against false theories and assertions, but to no avail. Having read all his works, one has to confess that Semmelweis’ sarcasm and bitterness might have been responsible for that: e.g., he calls the professors who do not follow his ideas “infectors” who should be “impeached” immediately:

*Nicht die Gebärhäuser müssen kassirt werden, um die Wöchnerinnen gesund zu halten, sondern sämmtliche Professoren der Geburtshilfe, welche Epidemiker sind, müssen kassirt werden, um die Wöchnerinnen gesund zu halten. *[…] *Wenn alle Professoren der Geburtshilfe, welche Epidemiker sind, mein Werk mit so wenig Nutzen lesen, wie Sie Herr Hofrath, dann ist freilich keine Hoffnung, daß das Menschengeschlecht von der Geißel des Kindbettfiebers früher befreit werde, als bis sämtliche Epidemiker ausgestorben. Aber das kostet noch unzähligen Wöchnerinnen das Leben, und wenn ich die Macht dazu hätte, und wenn ich keine andere Wahl hätte, als entweder noch unzählige Wöchnerinnen am Kindbettfieber, welche gerettet hätten werden können, sterben zu lassen, oder durch Kassirung sämmtlicher Professoren der Geburtshilfe, welche Epidemiker sind, und entweder meine Lehre nicht lernen wollen, oder meine Lehre nicht mehr lernen können, diese Wöchnerinnen zu retten, so würde ich die Kassirung der Professoren wählen, weil ich der Ueberzeugung bin, daß, wo es sich um die Verhütung der Ermordung Tausender und Tausender von Wöchnerinnen und Säuglingen handelt, ein paar Dutzend Professoren nicht in Betracht kommen.*^19^ (“It is not the maternity hospitals that have to be shut down to keep women in childbed healthy, all professors of obstetrics believing in epidemics have to be removed to keep women in childbed healthy. […] If all professors of obstetrics believing in epidemics are reading my book with as little profit as you do, *Herr Hofrath*, there is no hope that mankind will be freed from the scourge of childbed fever earlier, for all believers in epidemics will have died. Countless puerperae will die. And if I had the power and the choice between the death of innumerable women in childbed who could have been saved, or the removal from their posts of all professors of obstetrics who believe in epidemics and who do not want to follow my way or cannot even follow it, I would choose their removal, for I am deeply convinced that some dozens of professors are a *quantité négligeable *in comparison to thousands and thousands of murdered women and suckling infants.”)

Semmelweis frankly informs Siebold^20^ that he doubts his knowledge, that his pupils are not the guilty ones, since they have to trust their teacher, who unfortunately does not accept the truth. On the other hand, he begs him for respect and tells him that he knows him as a positive-minded, warm-hearted man who wants the best for his patients. To Scanzoni he writes that he was “right” for 13 years only because he remained silent.^21^

When Semmelweis’ book finally went to the printer in 1861, the scientific community did not accept it. This experience caused psychosomatic symptoms owing to his long-standing and deeply felt disappointment: bad conscience tortured him. The same had been true for Gustav Adolph Michaelis,^22^ who committed suicide in 1848 after having understood that he was responsible for the deaths of many women, including that of his own cousin.

In the case of Semmelweis, it was even more complicated: he wanted to save all patients, and he had clear results proving his theory, but was hindered by conservative traditionalists, who openly opposed and—what made the affair even more unbearable—privately used his methods.^23^ This permanent stress destroyed his health: in 1865, his relatives (including his wife) and friends took him from Budapest to Vienna. He thought he would be spending some time relaxing, but in fact was led into a newly built asylum for the mentally ill, the *Niederösterreichische Landesirrenanstalt*, in Lazarettgasse. When he realized what was going on, he tried to escape. Badly abused, he died from sepsis caused by open wounds and a dirty straightjacket 2 weeks later. It is unlikely that he was suffering from neurosyphilis, a risk to which gynecologists of the time were exposed.^24^ The same is true for presenile Alzheimer’s disease. Silló-Seidl seems to have found a more probable option based on documents and eyewitnesses:^25^ Semmelweis may have suffered from burnout, an illness unknown in the 19th century, combined with diabetes, a reasonable explanation for his insatiable thirst and his potency problems. Furthermore, his wife felt ashamed by her husband’s (rather rude) behavior. The combination of these factors and symptoms finally killed him, left alone in a cell, abandoned to indignity and dying from a secondary illness whose causes he had discovered.

At nearly the same time in the British Isles, Joseph Lister, the “father of antiseptic surgery,” had achieved his first successful results inspired by Louis Pasteur, and he admired Semmelweis.^26^ As early as in 1843, the American physician Oliver Wendell Holmes had success with disinfection and had to face the same enmity as Semmelweis.^27^ Evidently, there was no transatlantic exchange between the two men. Decades went by before the scientific community paid tribute to him. Until then, the majority slandered him as someone who fouled his own nest (*Nestbeschmutzer*). It was only after his death (quite a typical Austrian fate) that people called him “savior of the mothers” (*Retter der Mütter*).

One scene shows the prejudice he had to contend with: when he quoted the oath of the midwives in a medical meeting, the majority of those present did not understand the deeper sense of his choice of this text, but expected a scientific paper and thought he had gone mad. However, Semmelweis—with all his subtlety—wanted to show the ethical principle governing midwives and physicians.

## Semmelweis’ multilingual correspondence as a sign of individuality

Whatever the case may be, Semmelweis had always been somehow different—even in his doctoral thesis, which opened the gates to clinical practice for him, after having demonstrated his broad intellectual knowledge in a field that was not primarily medical.

Furthermore, he was multilingual. His first biographer^28^ documents his correspondence with Charles Henry Felix Routh, a former Viennese student, who became a famous physician. Routh wrote in Latin, Semmelweis answered in English:

*‘Pesth, 22/5/1861. Dear Friend,—As you have been so friendly in the year 1848* *at the meeting of the Englisch Doctors in London to bring forward a discussion on my opinion about the origin and prevention of fever in childbed, I take the liberty, having just finished a complete work upon the same subject, to send it you with the request to mention it again at the same meeting. I have also send my work to Webster, Copeland, Murphy, Simpson, Weber. Thanking you before for your trouble and hoping to hear something of you very soon, Your sincere friend. Ignaz Semmelweis.’* […] *Routh* […] *propagated Semmelweis’ teaching in England and wrote to him* […] *on January 23, 1849. ‘Comitiis in ultimis septimanis Novembris *(1848)* convocatis, illic discursus, in quo tuam inventionem enunciavi, reddens tibi, ut voluit Justitia, maximam gloriam, praelectus fuit. Enim vero possum dicere, totum discursum optime exceptum fuisse, et multi inter socios doctissimos attestaverunt argumentum convincens fuisse. Inter hos praecipue Webster, Copeland et Murphy, vires and *[*sic*!] *doctores clarissimi, optime locuti sunt. In Lancetto *[= *The Lancet*]*, Novembris 1848 possis omnia de hac* *controversia contingentia legere. Credisne novos casus, qui in hospitio ex tempore mei abitus admissi sunt, opinionem tuam confirmant? Febris ne puerperalis rarior est quam antea? Si morbus sic periculosus in cubilibus obstetriciis non adsit ut ante, certe effectus magni momenti denuo firmatus. In Praga quoque, ubi febris puerperalis tum frequenter obvenire solebat, eisdem causis consecuta fuit ingenerari!’ *(“In the meeting that took place in the last week of November, the paper in which I presented your discovery and showing greatest respect to you—what a sign of justice!—was read in public. I can even say that the whole discourse was accepted in the very best way, and so many of the most learned men attested that your argumentation was really convincing—especially Webster, Copeland and Murphy, most learned men and doctors, spoke in a very positive way. In the ‘Lancet’ of November 1848 you can read everything on the controversy. Do you think that new cases happening in the hospital after I left confirm your opinion? Isn’t the childbed fever rarer than before? If the so dangerous malady in maternity units is less present than before, the effect of high importance is proved again. Even in Prague, where so many cases of childbed fever occurred, it consequently arose from the same reasons!”) *Next follows a letter dated *[…] *21 May 1849: ‘Meas annotationes de tua inventione in libellulo publicavi.’ *(“I have published my annotations on your discovery in a booklet.”) […] *There is a third letter dated* […] *3 December 1849: ‘Jam inventionis tuae fama ac veritas in existimatione publica accrescit, et inter omnes medicorum societates quam res est maxime utilis, percipient et agnoscunt, nec vero etiam temere, nam magna est veritas et praevalebit.’ *(“The fame and verity of your discovery is already increasing in the public opinion, and in all medical societies they will perceive it as a most useful thing and – certainly not by chance – acknowledge it as an important truth that will last.”) […] *Routh was the first apostle of his teaching in the United Kingdom.*

These documents clearly show that (from the early stage of the discovery until its propagation and spreading) not everybody was against Semmelweis, but as a result of his personality, he was unable to see the positive side—neither in the late 1840s nor in the (early) 1860s, nor in between.^29^ Instead, he focused on the negative elements. Even an encouraging letter from his colleague Louis Kugelmann failed to console him (10.08.1861): *Nur sehr wenigen war es vergönnt, der Menschheit wirkliche, große und dauernde Dienste zu erweisen, und mit wenigen Ausnahmen hat die Welt ihre Wohltäter gekreuzigt und verbannt. Ich hoffe also, Sie werden in dem ehrenvollen Kampfe nicht ermüden, der Ihnen noch übrig bleibt.*^30^ (“To give real, great, and everlasting things to mankind was granted to very few, and except for a very small number the world crucified and banned its benefactors. I hope you will not tire during the remainder of your honorable fight.”)

## Semmelweis’ afterlife or: an insight into reception studies concerning a tragic hero

This personal tragedy is the reason why he is so inspiring for authors, directors and artists: in the final scene of Hans José Rehfisch’s drama *Doktor Semmelweis* (1934), the protagonist cuts himself with a contaminated scalpel and utters his final words, his *ultima verba*:^31^*Meine Damen und Herren! Wenn Sie Ihr Augenmerk auf den weitern Verlauf wenden wollen, werden Sie den etwa zwei Wochen dauernden Zerfall meiner körperlichen Existenz beobachten. Wenn ich Glück habe, lernt ihr daraus. *(“Ladies and Gentlemen! If you would pay attention to what happens next, you will observe the decline of my bodily existence over a period of approximately two weeks. If I am lucky, you will learn from it.”) Curtain. This invented literary moment has its roots in the theory that Semmelweis suffered his lethal sepsis during an operation: he was also a pioneer in gynecological surgery.^32^ Rehfisch’s dialogues are similar to Arthur Schnitzler’s *Professor Bernhardi* (1912 [26]), but even more disturbing, since Rehfisch’s tragic hero is a real person, not a fictional character.

Fred Zinnemann directed the short film *That Mothers Might Live *(1938), which won an Oscar. *Retter der Mütter *(1950), starring Karl Paryla, was produced in the German Democratic Republic, and Michael Verhoeven was responsible for *Arzt der Frauen *(1987–1988), starring Heiner Lauterbach. These movies show the fate of a man who was in the wrong place (the dangerous, conservative, nationalistic and anti-Semitic academic community of Vienna) at the “wrong” time (around the revolutionary year 1848), with the “wrong” political orientation (democratic and open-minded), the “wrong” roots (Hungarian born), and a Jewish-sounding name. He had mentors, but no chance—never.

Even as the hero of a children’s book, poor Semmelweis is a victim. In 1967, Willy Miksch, who also published on the Viennese *Laboratoriumspest* of 1898,^33^ portrays him as follows:

*Der Arm schmerzt ihn. Er streift die Ärmel hinauf, die Wunde ist tiefrot entzündet. „Da ist er wieder* *–* *mein Feind – der Mörder“, haucht der Arzt, und ein müdes Lächeln huscht über sein Gesicht. In diesem Augenblick merkt er, dass er allein ist. Ahnungsvoll geht er zur Türe. Sie ist verschlossen. Hilflos blickt er sich um: er sieht das vergitterte Fenster, das festgeschraubte Bett, den ganzen kahlen Raum – und er versteht. Die Hände schlägt er vor’s Gesicht und fängt haltlos zu weinen an. Langsam wird es Nacht. Auf dem Gang versieht ein Wärter seinen Dienst und blickt durch ein Guckloch in der Tür in jedes Zimmer. Immer noch sitzt Dr. Semmelweis auf dem Bett und weint. So hilflos allein, so einsam verlassen kann ein Mensch werden. Am 13. August 1865 ist Dr. Semmelweis im 47. Lebensjahr gestorben. Niemand hat seine Wunde bemerkt – und der Tod kommt so schnell, wenn man Leichengift in sich trägt. Der Feind hat sich gerächt, aber als er Dr. Semmelweis, seinen erbittertsten Bekämpfer, niederstreckt, da war dieser Arzt schon Sieger, die Welt hörte schon auf sein Wort. Nach wenigen Jahren nannten ihn die Bücher: Den Retter der Mütter!* (“His arm hurts. He is turning up his sleeves. The wound is deeply red and inflamed. ‘There he is again, my enemy, the murderer,’ the doctor whispers, accompanied by a tired smile crossing his face. In this very moment, he realizes that he is alone. Knowingly, he goes to the door. It is locked. Helpless, he looks around and sees the window with the grid, the bed fixed to the floor, the whole bare room—and he understands. He puts his hands in front of his face and weeps … Slowly, night falls. Outside, a guard is on his way and looks into this room and all the others through the peepholes in the doors. Dr Semmelweis is still sitting on his bed weeping. So helpless, so lonesome can a man be abandoned. On 13th of August 1865 Dr Semmelweis died in the 47th year of his life. Nobody had noticed his wound—and death comes quickly when corpse poison is within. The enemy took revenge, but Dr Semmelweis, the most prominent fighter against him, was victorious when struck down by him. The world was following his word. A few years later, the books called him savior of the mothers.”)

In contrast, one of Semmelweis’ modern biographers finds him, at least partly, responsible for his fate:^34^

*Viele Semmelweis-Biographen stilisieren ihn zum tragischen Helden à la Aischylos, der von böswilligen Göttern zerstört wird. Doch in Wahrheit paßt er eher in eine Tragödie von Sophokles, in der das Schicksal des Helden nicht von den Handlungen der Götter bestimmt wird, sondern durch einen tragischen Fehler in seinem eigenen Wesen. Ignaz Semmelweis steuerte, nachdem er seine Mission gefunden hatte, unweigerlich auf sein tragisches Ende zu. Genau so hätte Sophokles die Geschichte wohl angelegt, mit einem Chor sterbender Mütter als Hintergrund: großer Held, große Wahrheit, große Mission, dazu Wahnsinn und Überheblichkeit, die zum Ruin führen. Die Götter, sprich Professoren der Gynäkologie, waren nicht schuld, sondern der Held selbst. *(“Many biographers depict him as a tragic hero à la Aischylos, destroyed by evil gods. However, he fits better into a Sophoclean tragedy, where the hero’s fate is not determined by the gods, but by a tragic flaw in his own character. After having found his mission, Ignaz Semmelweis inevitably headed towards his tragic destiny. Precisely like this would Sophocles have written the plot—including a choir of dying women in the background: a great hero, a great truth, a great mission, insanity, and hybris causing ruin. The gods, i.e., the professors of gynecology, were not guilty, but the hero himself.”)

Or, as Durnova put it: *Er hatte sein Lebensziel auf die Waschschüssel mit Chlorkalklösung gerichtet, und das merkte man ihm an. *[…] *Semmelweis wurde zu einem kranken Menschen, der in seinem Kampf um die Entdeckung Fehler machte. Gemeint sind damit menschliche Fehler, die seinen Niedergang unabdingbar machten. *[…] *Semmelweis war ein Außenseiter und sein Leben war alles andere als leicht gewesen. Und dies galt scheinbar auch für seinen Kampf um die Wahrheit.*^35^ (“The focus of his life was a jug of chlorinated lime solution, and everybody noticed. […] Semmelweis became an ill man who made mistakes while fighting for his discovery. His mistakes were human, causing his ruin. […] Semmelweis was an outsider, and his life had been anything but easy. And this seemed to be true for his fight for the truth as well.”)

Nevertheless, the stubborn gynecologist would have been very pleased that, in 1967, the rebellious sculptor Alfred Hrdlicka created a lasting monument to him—in the courtyard of the University of Vienna, the place of his enduring triumph and personal ruin. However, even this belated honor did not work as planned: the monument was intended to be unveiled in 1965, when the university was 600 years old and Semmelweis dead for a century; however, those in charge failed to take into consideration the amount of time Hrdlicka would need to realize his project.^36^

## Footnotes

1 Semmelweis [1].

2 Semmelweis [2].

3 Weis [3]. Schönberger [4]. Djakovic A.; Dietl J. [5].

4 http://semmelweis200.hu and http://semmelweismuseum.hu mention the title.

5 Durnova [6].

6 All (sometimes rather free) translations are made by the author of this article. My warmest thanks go to my colleague Danuta Shanzer for polishing my English. All remaining errors are mine. Furthermore, I am deeply grateful to the anonymous reviewers for their suggestions for improvement.

7 Céline [7]. Cf. Céline [8, p. 25]: *Während dieser Krise in seinem Beruf treibt er sich gern in den botanischen Gärten herum, wo er den im Preisen der Vorzüge einfacher Gewächse unerschöpflichen Pflanzenkenner Bozatov zu Rate zieht. **Die durchwegs empirische Wissenschaft dieses Kräuterkundigen entzückt ihn.* (“Facing problems in his job, he loves to strive through the botanical gardens, where he consults Bozatov, a mere specialist on plants praising the positive sides of simple herbs without end. He is delighted by the thoroughly empirical method of the herbalist.”).

8 Helander [9].

9 Cf. http://www.deutschestextarchiv.de/book/view/blumenbach_naturgeschichte_1816?p=442. Semmelweis uses modern secondary literature in vernacular language translating it into Latin in his thesis, e.g., when he discusses the different kinds of metamorphoses in plants (p. 20): […] *Hayne tradit Bryophyllum calycinum in India vegetans, mane acido, vespere amaro gustum adficere, dum sub meridie sapore careat, idem probat colorum in certis plantis mutatio.* (“Hayne informs us that the B.c. growing in India has an acid taste in the morning and a bitter one in the evening, while having no taste at all at noon; the same is true for the change of colors in certain plants.”) Cf. Heyne [10].

10 Lesky [11].

11 Semmelweis [1, p. 53–4].

12 As part of the *Neuburger Lesky Bibliothek*, named after Max Neuburger and Erna Lesky, Semmelweis’ texts can be used in the *Josephinum* or read online: https://ub.meduniwien.ac.at/blog/?tag=retter-der-muetter.

13 Wilson [12]. Medicus [13].

14 Semmelweis [14, p. 53]: *‘anno currente plusculae feminae Hafnienses vel abortum passae, vel difficultate partus mortuos ediderunt, vel sectione per chirurgum sibi extrahi discerpique viderunt, vel febre variolisque exstincte. **Et pleraeque femellas ediderunt, imbecillitatis indicio. Juvit humida anni constitutio et frigida, qua laxata uteri ligamenta foetum, ut decet, constringere non potuerunt.’ *(“In the current year, some women from Copenhagen suffered from a miscarriage, after some difficulties delivered dead babies, had to undergo a chirurgical extraction and discerption, or died from fever and smallpox. In addition, many gave birth to girls as a sign of weakness. The wet and cold constitution of the year supported this situation; the widened tissues of the uterus could not hold the fetus as they should have done.”): https://archive.org/details/offenerbriefsm00semm/page/n8. When the president of the *Société d’Histoire de la Naissance*, Marie-France Morel, talked about “Midwives in History” and “Research Paths” in Vienna (13.05.2019), she stressed the fact, that for centuries women were put close to open fire while giving birth, because cold temperature was seen as mortal danger.

15 Semmelweis [15, p. 12–4]: *Sie opfern einzelne Wöchnerinnen der cadaverösen Infection, um desto mehr Wöchnerinnen übrig zu behalten zur Ermordung durch epidemische Einflüsse, und durch andere ätiologische Momente, welche Sie aufzählen, und welche wir beurtheilen werden. *[…] *Es wäre mir nur angenehm, wenn meine Gegner etwas antworten würden, denn würden Sie etwas anderes antworten als „peccavi“, so würde ich nur erneuerte Gelegenheit haben, meiner Lehre zu einem glänzenden Siege zu verhelfen. Herr Hofrath sagen, daß ich in maßloser Weise gegen Alle, die nicht in meiner Meinung sind, oder die auch nur Zweifel über dieselbe zu äußern wagten, zu Felde gezogen sei. *[…] *Aber nicht meiner Meinung zu sein, ist gleichbedeutend mit „ein Mörder sein.“*: http://digital.onb.ac.at/OnbViewer/viewer.faces?doc=ABO_%2BZ219438009. (“You are sacrificing single women in childbed to the cadaverous infection, for having more puerperae left to be murdered by epidemic influence and other etiological moments you are listing and which we will evaluate. […] It would be pleasant, if my opponents gave any answer at all, because if you answered something other than ‘I’m a sinner’, I would have another chance to promote my doctrine as bright and beaming winner. You say, *Herr Hofrath*, that I fought without any restrictions against all people, who do not believe what I do or who doubt what I do […] but, not being on my side means being a murderer.”)

16 Semmelweis [16, p. 20–1]: *Sollten Sie aber, Herr Hofrath, ohne meine Lehre widerlegt zu haben, fortfahren, Ihre Schüler und Schülerinnen in der Lehre des epidemischen Kindbettfiebers zu erziehen, so erkläre ich Sie vor Gott und der Welt für einen Mörder, und die „Geschichte des Kindbettfiebers“ würde gegen Sie nicht ungerecht sein, wenn selbe Sie, für das Verdienst der Erste gewesen zu sein, der sich meiner lebenrettenden Lehre widersetzt, als medicinischen Nero verewigen würde*: https://reader.digitale-sammlungen.de/de/fs1/object/display/bsb10912458_00001.html. (“If you, *Herr Hofrath*, without having refuted my findings, continue teaching your pupils the doctrine of epidemic childbed fever, I declare you—by God and in presence of the whole world—as a murderer, and the ‘history of childbed fever’ would not be unjust, if it immortalized you, the first one who was against my life-saving methods, as a medical Nero.”)

17 Standing alone against the rest of the world, he was called a medical “Don Quijote”: https://www.spektrum.de/news/der-retter-von-der-traurigen-gestalt/1574034.

18 Semmelweis [15, p. 9;16].

19 Semmelweis [15, p. 20–1]. [Cf. Semmelweis [16, p. 6;8]: *Ich trage in mir das Bewußtsein, daß seit dem Jahr 1847 tausende und tausende von Wöchnerinnen und Säuglinge gestorben sind, welche nicht gestorben wären, wenn ich nicht geschwiegen, sondern jedem Irrthume, welcher über Puerperal-Fieber verbreitet wurde, die nöthige Zurechtweisung hätte zu Theil werden lassen* […]*. Für mich gibt es kein anderes Mittel, dem Morden Einhalt zu thun, als die schonungslose Entlarvung meiner Gegner, und Niemand, der das Herz auf dem rechten Fleck hat, wird mich tadeln, daß ich dieses Mittel ergreife.* (“Deep inside me I know that, from 1847 onwards, thousands and thousands of puerperae and babies have died who would not have died if I had not been silent, but castigated every single mistake published on childbed fever […]. In my opinion there is no other remedy against this homicide than to recklessly unmask my opponents, and nobody, having his heart in the right place, will castigate me for acting like this.”)].

20 Semmelweis [15, p. 27].

21 Semmelweis [15, p. 40].

22 Semmelweis [16, p. 19]: *Meine Lehre basirt darauf, daß Michaelis schmerzlichen Angedenkens, meine Lehre im Gebärhause zu Kiel bestätigt gefunden.* (“My doctrine is based on the fact that Michaelis—what a painful memory!—found the proof in the maternity hospital in Kiel.”)—Semmelweis [2, p. 65]: *Indem Professor Rokitansky seit 1828 an der pathologisch-anatomischen Anstalt fungirt, so konnten theils aus seiner Erinnerung, theils aus den Sectionsprotokollen, so wie durch Einvernehmen anderer Aerzte, diejenigen Assistenten und Studirenden hervorgesucht werden, die sich mit Leichenuntersuchungen befasst haben, und es hätte sich ergeben, ob die Zahl der Erkrankungen in der Gebäranstalt mit der Verwendung der Assistenten und Studirenden in der Sectionskammer in Zusammenhang stand. Die Commission durfte* […] *auf höheren Befehl ihre Aufgabe nicht lösen. Consequent meiner Ueberzeugung muss ich hier das Bekenntniss ablegen, dass nur Gott die Anzahl derjenigen kennt, welche wegen mir frühzeitig ins Grab gestiegen. Ich habe mich in einer Ausdehnung mit Leichen beschäftigt, wie nur wenige Geburtshelfer. *(“Because Professor Rokitansky had been the head of the pathological-anatomical institute from 1828 on, out of his memory, due to section protocols, and talking to other physicians, those assistants and students who worked with dead bodies could be found. It would have become evident if there had been a connection between the number of diseases in the maternity hospital and the use of assistants and students in the morgue. The commission was not allowed—because of higher order—to continue its work. Following my persuasion, I have to confess that only God knows the number of those who had to step much too early into their graves—because of me … I worked with dead bodies to such an extent as only a few of obstetricians did.”)

23 Maisel [17, p. 102]: *Trotz Unterstützung durch fortschrittliche Professoren […] wurde sein Vertrag in Wien nicht verlängert, seine Methode der Chlorwaschung jedoch beibehalten (‘Warten und Waschen’), wodurch die Sterblichkeit im Kindbett deutlich reduziert werden konnte*. (“Despite the support of progressive professors his contract in Vienna was not prolonged. However, his method of washing hands (‘wait & wash’) with chlorinated lime solution was kept; therefore, significantly fewer women died in childbed.”).

24 Bankl ^3^[18, p. 81–98] = Ignaz Philipp Semmelweis. Gehirnerkrankungen waren eine häufige Komplikation der Syphilis.

25 Silló-Seidl [19]. Silló-Seidl [20].

26 Zoltán I. https://www.britannica.com/biography/Ignaz-Semmelweis: *I think with the greatest admiration of him and his achievement and it fills me with joy that at last he is given the respect due to him.*

27 https://www.ncbi.nlm.nih.gov/pmc/articles/PMC2866610/ (10.2105/AJPH.2009.185363).

28 Schürer [21, p. 43–4] & Obituary. Charles Henry Felix Routh [22].

29 Semmelweis [14, p. 27]:* Von der großen Anzahl der Professoren der Geburtshilfe haben innerhalb fünfzehn Jahren nur zwei die von mir entdeckte Wahrheit erkannt, selbe mit Erfolg beobachtet, und nur diese zwei waren zugleich auch redlich genug, das auch öffentlich anzuerkennen; Einer dieser Professoren der Geburtshilfe war Michaelis in Kiel, der andere ist der Geh. **Hofrath Prof. Dr. Lange in Heidelberg. *(“Out of the huge number of professors of obstetrics in 15 years only two have seen the truth discovered by me and observed it with success, and only the two of them were honest and of sufficient integrity to acknowledge it in public. One was Michaelis in Kiel, the other is the *Geheime Hofrath Prof Dr *Lange in Heidelberg.”)

30 Ibid.:V.

31 Rehfisch [23, p. 393–502] = *Doktor Semmelweis*. Cf. Picard [24], where Semmelweis’ friend Marcojevisc tries to calm him:73–4: *Ich würde sie so rasch nicht Mörder nennen, denn es erschwert dir, sie zu überzeugen. Nicht Hinz und Kunz sind deine Gegner, Ignaz, es sind geheime Räte, Professoren, die hohes Ansehn überall genießen. Bei den Kollegen und bei der Regierung. *[…] *Weil man mit Höflichkeit oft mehr erreicht!* *–* *Wie ich schon sagte: nahst du dich als Freund, wird Tür und Tor sich willig weit dir öffnen, und ohne Kampf kannst du die Burg erobern, wenn du geschickt von innen sie erklimmst. *[…] *Ich rate dir, laß größ’re Rücksicht walten und bändige den ungestümen Zorn. Wenn du dich wie ein Wütender gebärdest und um dich schlägst und schimpfst und spuckst und geiferst, hat man es leicht, dich einen Narr’n zu schelten und viele Gläubige dafür zu finden. Du kannst dich nicht benehmen wie ein Kutscher, willst du als ein Professor Achtung fordern. Und deine Gegner sind nicht alte Mähren, die mit der Peitsche du zu folgen zwingst. Du solltest lieber dich dazu bequemen, die Gunst der Herren für dich zu gewinnen. Laß hier und da ein Schmeichelwort vernehmen – kein Mensch ist frei von Selbstgefälligkeit: Man liebt den Spiegel, der in Glanz uns zeigt – so machst du sie dir rasch und leicht gewogen. Und weißt du deine Worte wohl zu setzen, wirst du für deine Lehre auch Beachtung finden. *(“I would not call them murderers so quickly. It makes it more difficult to convince them. Your opponents are not Tom, Dick and Harry, Ignaz, but counsellors, professors, highly renowned people—by their colleagues, the government, everywhere. […] Quite often, you will have success by politeness. As I said before: coming as a friend, the doors will be open wide, you can conquer the castle without any fight, climbing to it cleverly from inside. […] I beg you, be more careful and master your immoderate fury. If you are acting furiously and offensively, kicking, hissing, and spitting, it will be so easy to call you a lunatic and to find many sharing this opinion. You cannot behave like a coachman if you want to get respect as a professor. In addition, your opponents are no shabby nags that shall follow you forced by a whip. You should better work on gaining the friendship of these gentlemen. Try to be charming—nobody dislikes that. Everybody loves the mirror showing and reflecting his brightness—so, everybody will be—quick and easy—on your side. And if you manage to find the right words, they will accept your doctrine.”) In both plays, the dialogues are based on Semmelweis’ writings, which make them vivid and realistic.

32 Lesky [25, p. 93–5].

33 Miksch [27, p. 31]. Miksch [28].

34 Nuland [29, p. 191].

35 Durnova [6, p. 117]. Semmelweis [14, p. 36–7]: *Sollten sich die Professoren nicht baldigst dazu bequemen, ihre Schüler und Schülerinnen in meiner Lehre zu unterrichten, sollten die Regierungen noch länger die Kindbettfieber-Epidemien in den Gebärhäusern dulden, so werde ich, um wenigstens die in geographischen Verbreitung Entbindenden vor dem Kindbettfieber zu schützen, mich an das hilfsbedürftige Publikum wenden, ich werde sagen: Du Familienvater weißt Du, was das heißt, einen Geburtshelfer oder eine Hebamme zu Deiner Frau zu rufen, welche bei der Geburt eines Beistandes benötigt, das heißt so viel als Deine Frau und Dein noch ungeborenes Kind einer Lebensgefahr aussetzen. Und wenn Du nicht Wittwer werden willst, und wenn Du nicht willst, daß Deinem noch ungeborenen Kinde der Todeskeim eingeimpft werde, und wenn Deine Kinder ihre Mutter nicht verlieren sollen, so kaufe Dir um einige Kreuzer einen Chlorkalk, gieße ein Wasser darauf, und lasse den Geburtshelfer und die Hebamme Deine Frau ja nicht innerlich untersuchen, bevor sich nicht der Geburtshelfer, bevor sich nicht die Hebamme in Deiner Gegenwart die Hände in Chlor gewaschen haben, und auch dann noch laß den Geburtshelfer und die Hebamme noch nicht innerlich untersuchen, bis Du Dich durch Betasten derer Hände überzeugt hast, daß sich der Geburtshelfer und die Hebamme so lange gewaschen haben, daß die Hände schlüpfrich geworden. Aber deshalb darfst Du die Schuld nicht dem Geburtshelfer, nicht der Hebamme zuschreiben, daß selbe für Deine Frau lebensgefährlich sind, die Schuld trägt der Professor der Geburtshilfe, bei welchem der Geburtshelfer, die Hebamme Geburtshilfe gelernt, und welcher Professor dem Geburtshelfer, der Hebamme nicht gelehrt, das verhütbare Resorbtionsfieber in der Fortpflanzungsperiode des Weibes, entstanden durch verhütbare Infection von Außen, zu verhüten. Ich hoffe, das hilfebedürftige Publicum wird gelehriger sein, als die Professoren der Geburtshilfe. *(“If the professors won’t inform their pupils as soon as possible about my doctrine, should the governments endure the epidemics of childbed fever any longer, I will address the public that needs help to protect the women giving birth nearby. I will say: Father of your family, do you know what it means to call an obstetrician or a midwife to your wife who needs assistance while giving birth? It means to put your wife and your unborn child into mortal danger. And if you do not want to become a widower and if you do not want the germ of death to be implanted in your unborn baby, and if your children are not to lose their mother, buy—for a small amount of money—chlorinated lime solution, pour water on it, and do not allow the obstetrician or midwife to undertake an internal examination of your wife until they have washed their hands in your presence, and forbid them to make an internal examination before you have touched their hands and you feel that they have washed them until the hands became slippery. However, you must not declare the obstetrician or the midwife guilty, but the professor who taught them and who did not teach them that the preventable resorption fever during the reproductive period of the woman, caused by a preventable infection from outside, can be prevented. I do hope that the public needing help will learn more than the professors of obstetrics.”).

36 Maisel [17, p. 102]: *Relief von Alfred Hrdlicka, beschafft von der II. Universitäts-Frauenklinik, enthüllt 1967*: http://semmelweis.info/hello-hand-hygiene-community-would-you-recognize-the-person-on-this-relief-without-nameline/.
